# Efficacy and Safety of Lorundrostat in Patients With Uncontrolled and Treatment-Resistant Hypertension: A Systematic Review and Meta-Analysis

**DOI:** 10.7759/cureus.91681

**Published:** 2025-09-05

**Authors:** Shahmeen Rasul, Anurag Rawat, Maryam Mazhar, Daksh Kumar, Anusha Laxman, Sandipkumar S Chaudhari, Rana M Ahzam, Areeba Khan

**Affiliations:** 1 Trauma and Orthopaedics, Royal Free Hospital, London, GBR; 2 Interventional Cardiology, Himalayan Institute of Medical Sciences, Dehradun, IND; 3 Medicine, Services Institute of Medical Sciences, Lahore, PAK; 4 Internal Medicine, Bahria University Medical and Dental College, Pakistan Navy Ship (PNS) Shifa Hospital, Karachi, PAK; 5 Research, Chettinad Academy of Research and Education, Chennai, IND; 6 Cardiothoracic Surgery, University of Alabama at Birmingham, Birmingham, USA; 7 Family Medicine, University of North Dakota School of Medicine and Health Sciences, Fargo, USA; 8 Internal Medicine, CMH Lahore Medical College and Institute of Dentistry, Lahore, PAK; 9 Critical Care Medicine, United Medical and Dental College, Karachi, PAK

**Keywords:** aldosterone synthase inhibitor, lorundrostat, meta-analysis, systematic review, treatment-resistant hypertension

## Abstract

This systematic review and meta-analysis evaluated the efficacy and safety of lorundrostat, a novel aldosterone synthase inhibitor, in patients with uncontrolled or treatment-resistant hypertension. A comprehensive literature search was conducted across major electronic databases, including MEDLINE, Embase, Cochrane CENTRAL, Web of Science, and Scopus through July 25, 2025. Randomized controlled trials comparing lorundrostat to placebo in adult patients with uncontrolled hypertension were included. Three studies met the inclusion criteria and were analyzed using Review Manager 5.4.1 (The Cochrane Collaboration, London, England, UK) with random-effects models. The pooled analysis demonstrated that lorundrostat achieved a statistically significant reduction in systolic blood pressure of 8.04 mmHg compared to placebo (95% confidence interval (CI): -10.69 to -5.39) with low heterogeneity (I^2^ = 28%). Subgroup analysis revealed comparable efficacy between 50 mg and 100 mg doses, suggesting a relatively flat dose-response relationship within this range. This magnitude of blood pressure reduction is clinically meaningful and comparable to established fourth-line therapies like spironolactone. Regarding safety, lorundrostat was associated with a significantly higher risk of adverse events compared to placebo (risk ratio (RR): 1.47; 95% CI: 1.32 to 1.64), though most were mild to moderate in severity. Importantly, no significant difference was observed in serious adverse events (RR: 1.01; 95% CI: 0.39 to 2.63), providing reassurance about the overall safety profile. These findings suggest that lorundrostat represents a promising therapeutic option for treatment-resistant hypertension, offering clinically significant blood pressure reductions with an acceptable safety profile. However, long-term cardiovascular outcome studies and direct comparisons with existing therapies are needed to fully establish its clinical role.

## Introduction and background

Hypertension affects approximately 1.13 billion people worldwide and remains the leading cause of cardiovascular morbidity and mortality [1]. Despite the availability of multiple antihypertensive drug classes, achieving optimal blood pressure control remains challenging, with over 70% of hypertensive patients in the United States having uncontrolled blood pressure [2]. Treatment-resistant hypertension (TRH), defined as uncontrolled blood pressure despite concurrent use of three or more antihypertensive medications at maximally tolerated doses or controlled blood pressure requiring four or more medications, affects approximately 10-20% of all hypertensive patients [3,4]. The prevalence of apparent TRH has been estimated at 17.7% to 19.7% according to recent American Heart Association guidelines, representing approximately 10.3 million US adults [5].

The pathophysiology of TRH is complex and multifactorial, with dysregulated aldosterone production playing a central role in many cases [6]. Aldosterone, a mineralocorticoid hormone synthesized in the zona glomerulosa of the adrenal cortex, regulates sodium and water homeostasis through activation of mineralocorticoid receptors (MRs) in the kidney [7]. Excessive aldosterone production, whether from primary aldosteronism or obesity-related aldosterone dysregulation, contributes significantly to hypertension pathogenesis by promoting sodium retention, vascular inflammation, and cardiac fibrosis [8,9]. Primary aldosteronism accounts for at least 5-10% of all hypertensive cases and up to 20% of patients with TRH [10,11].

Current therapeutic approaches for aldosterone-mediated hypertension primarily rely on MR antagonists (MRAs) such as spironolactone and eplerenone [12]. Aldosterone synthase (CYP11B2) represents an attractive therapeutic target as it catalyzes the final three enzymatic steps in aldosterone biosynthesis from 11-deoxycorticosterone [13,14]. Unlike MRAs, aldosterone synthase inhibitors (ASIs) prevent aldosterone production at its source, potentially avoiding the compensatory increase in aldosterone levels and reducing both genomic and non-genomic aldosterone effects [15].

Lorundrostat (MLS-101) represents a novel, highly selective ASI with a selectivity ratio of 374:1 for CYP11B2 over CYP11B1 inhibition [16]. Recent phase II clinical trials, including the Target-HTN and Advance-HTN studies, have demonstrated lorundrostat's efficacy in lowering blood pressure in patients with uncontrolled hypertension, with particularly robust effects in patients with obesity and suppressed renin activity [17,18]. The drug appears to be well-tolerated, with manageable adverse events (AEs), primarily hyperkalemia and modest declines in estimated glomerular filtration rate [19].

Given the pressing clinical need for effective treatments for TRH and the promising early clinical data, a comprehensive systematic review and meta-analysis of lorundrostat's efficacy and safety profile is warranted to inform evidence-based clinical decision-making and guide future therapeutic strategies for this challenging patient population. The aim of this study was to assess the efficacy and safety of lorundrostat in subjects with uncontrolled or TRH.

## Review

Methodology

This systematic review and meta-analysis were conducted as per the guidelines of Preferred Reporting of Systematic Review and Meta-Analyses (PRISMA).

Literature Search and Search Strategy

An extensive and systematic search of the literature was undertaken across several major electronic databases to identify studies assessing the efficacy and safety of lorundrostat in individuals with uncontrolled or TRH. The search strategy included MEDLINE (via PubMed), Embase, the Cochrane Central Register of Controlled Trials (CENTRAL), Web of Science, and Scopus, covering all records from the inception of each database up to July 25, 2025. To maximize the retrieval of relevant data, no restrictions were applied regarding language or date of publication.

The search strategy was developed using a combination of Medical Subject Headings (MeSH) terms and free-text keywords. The primary search terms included: "lorundrostat," "MLS-101," "aldosterone synthase inhibitor," "CYP11B2 inhibitor," combined with terms related to hypertension such as "hypertension," "blood pressure," "treatment-resistant hypertension," "uncontrolled hypertension," and "resistant hypertension." Boolean operators (AND, OR) were used to combine search terms appropriately. The complete search strategy was adapted for each database according to its specific syntax and controlled vocabulary.

Additional searches were conducted in clinical trial registries, including ClinicalTrials.gov, World Health Organization International Clinical Trials Registry Platform (WHO ICTRP), and the European Union Clinical Trials Database (EudraCT), to identify ongoing or unpublished studies. Reference lists of included studies, relevant systematic reviews, and clinical practice guidelines were manually screened to identify any additional eligible studies not captured by the electronic search. Conference abstracts from major cardiovascular and hypertension meetings were also reviewed for potentially relevant studies. Search was performed by two authors. Any disagreement was resolved through discussion.

Study Selection and Eligibility Criteria

Study selection was performed independently by two reviewers using a two-stage screening process. Initial screening was conducted based on titles and abstracts, followed by full-text review of potentially relevant articles. Disagreements between reviewers were resolved through discussion or consultation with a third reviewer when consensus could not be reached.

Studies were considered eligible for inclusion if they met the following criteria: (1) Population: adult patients (≥18 years) with uncontrolled hypertension or TRH; (2) Intervention: treatment with lorundrostat (MLS-101) at any dose; (3) Comparator: placebo, active control, or standard care; (4) Outcomes: reporting of systolic blood pressure (SBP) changes, AEs, or serious adverse events (SAEs); (5) Study design: randomized controlled trials (RCTs) or observational studies. Studies were excluded if they were case reports, case series, or observational studies; (4) did not report extractable data on predefined outcomes; (5) were duplicate publications or multiple reports of the same study population, in which case the most comprehensive or recent publication was included.

Data Extraction and Outcomes

Two reviewers independently carried out data extraction using a pre-tested and standardized form. Information collected from each eligible study included: study details (first author, year of publication, design, duration, and number of participants); baseline characteristics of participants (age and sex); and specifics of the intervention (lorundrostat dosage). The main efficacy outcome was the change in SBP, reported in mmHg, from baseline to the end of the study. Safety outcomes included the incidence of AEs and SAEs.

Quality Assessment

Two reviewers independently evaluated the methodological quality of the included randomized trials using the updated Cochrane Risk of Bias tool (RoB 2) [20]. This evaluation encompassed five key areas: (1) potential bias related to the randomization process; (2) bias resulting from deviations from the intended interventions; (3) bias associated with missing outcome data; (4) bias in outcome measurement; and (5) bias stemming from selective reporting of results. Each domain was classified as presenting a low risk of bias, some concerns, or a high risk of bias. An overall judgment regarding the risk of bias was then assigned to each study based on these domain-level assessments.

Data Analysis

All statistical analyses were conducted using Review Manager (RevMan) version 5.4.1 (The Cochrane Collaboration, London, England, UK). For continuous variables such as changes in blood pressure, mean differences (MDs) along with 95% confidence intervals (CI) were calculated. For categorical outcomes, including AEs, risk ratios (RR) with corresponding 95% CI were employed. Heterogeneity among studies was evaluated using both the I^2^ statistic and Cochran’s Q test. Interpretation of I^2^ values was as follows: 0-40% may indicate minimal heterogeneity, 30-60% moderate heterogeneity, 50-90% substantial heterogeneity, and 75-100% considerable heterogeneity. A random-effects model was applied in all cases to account for potential variability across studies. We were unable to perform a publication bias assessment as the number of included studies was less than 10.

Results

**Figure 1:**
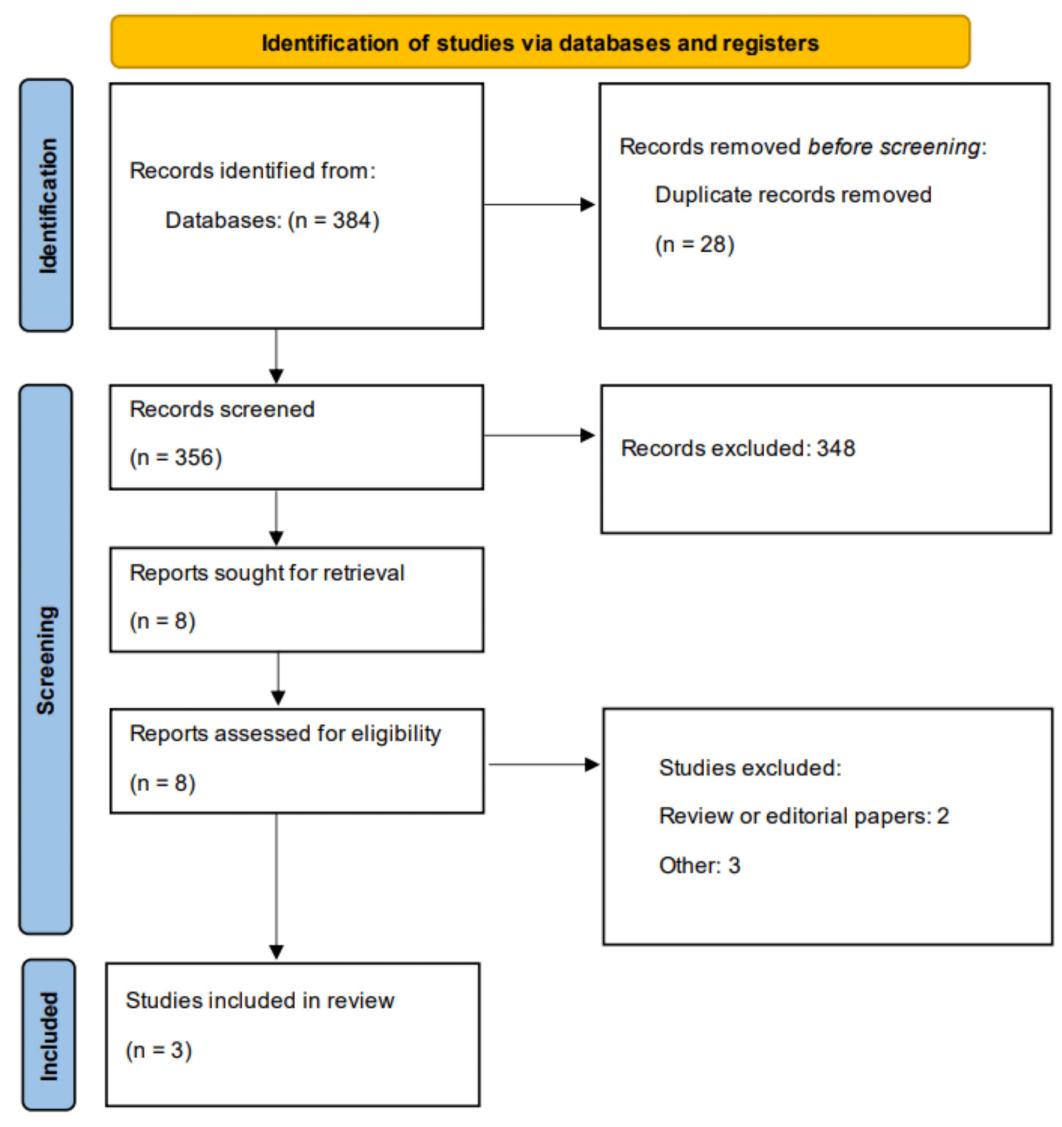
Study selection process (PRISMA flowchart) PRISMA: Preferred Reporting of Systematic Review and Meta-Analyses

Online database searching yielded 384 studies. After removing duplicates, 356 studies were initially screened. Full text of eight studies was obtained, and based on detailed eligibility criteria, three studies were included in the meta-analysis. Figure 1 presents the detailed study selection process. Table 1 shows the characteristics of included studies. Table 2 presents the quality assessment of the included studies. All included studies have a low risk of bias.

**Table 1:** Characteristics of included studies

Author	Year	Groups	Sample Size	Dose of Lorundstat	Duration of Follow-Up	Age (Years)	Male (n)
Laffin et al. [16]	2023	Lorundrostat	28	50 mg	8 weeks	64.7	13
		Lorundrostat	30	100 mg		68.7	12
		Placebo	30			62.6	13
Laffin et al. [17]	2025	Lorundrostat	188	50 mg	4 weeks	61	110
		Placebo	95			59.1	62
Saxena et al. [18]	2025	Lorundrostat	541	50 mg	6 weeks	61.7	294
		Lorundrostat	270	100 mg		61.4	142
		Placebo	272			61.8	139

**Table 2:** Quality assessment of included studies

Study	Randomization Process	Deviations From Intended Interventions	Missing Outcome Data	Measurement of Outcome	Selection of Reported Results	Overall Risk of Bias
Laffin et al. (2023) [16]	Low	Low	Low	Low	Low	Low
Laffin et al. (2025) [17]	Low	Low	Low	Low	Low	Low
Saxena et al. (2025) [18]	Low	Low	Low	Low	Low	Low

Change in SBP from Baseline

**Figure 2:**
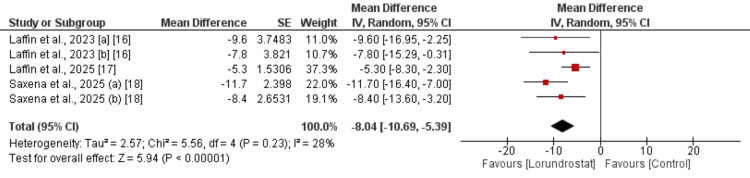
Effect of lorundostat on change in SBP [a] 50 mg dose; [b] 100 mg dose; red square indicates mean difference of individual study; black diamond indicates pooled estimate; SBP: systolic blood pressure References [16-18]

Three studies were included to compare the change in SBP from baseline. Two of these studies were included twice, as they assessed two different doses of lorundrostat separately. As shown in Figure 2, the reduction in SBP was significantly greater in participants receiving lorundrostat compared to those receiving placebo (MD: -8.04 mmHg; 95% CI: -10.69 to -5.39). Low heterogeneity was observed across the study results (I^2^ = 28%). A subgroup analysis based on lorundrostat dose is presented in Table 3. Both the 50 mg and 100 mg doses were associated with significant reductions in SBP, with no significant difference in effect size between the two groups. Heterogeneity remained low in both subgroups.

**Table 3:** Subgroup analysis RR: risk ratio; CI: confidence interval; SBP: systolic blood pressure; NA: not applicable * presented as mean difference.

Outcomes	Dose of Lorundstat	RR (95% CI)	I^2^
Change in SBP*	50 mg	-8.44 (-12.97 to -3.91)	30%
	100 mg	-8.20 (-12.48 to -3.93)	0%
Adverse events	50 mg	1.43 (1.25 to 1.64)	0%
	100 mg	1.54 (1.28 to 1.84)	0%
Serious adverse events	50 mg	1.41 (0.28 to 7.15)	63%
	100 mg	0.63 (0.21 to 1.90)	NA

Adverse Events (AEs)

**Figure 3:**
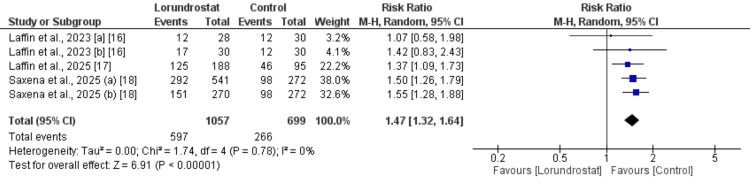
Comparison of adverse events between two groups [a] 50 mg dose; [b] 100 mg dose; blue square indicates risk ratio reported by individual study; black diamond indicates pooled estimate References [16-18]

Three studies were included to assess the risk of AEs, and the results of the pooled analysis are presented in Figure 3. The analysis showed that the risk of AEs was significantly higher in participants receiving lorundrostat compared to those receiving placebo (RR: 1.47; 95% CI: 1.32 to 1.64). No significant heterogeneity was observed among the study results (I^2^ = 0%). Subgroup analysis by dose revealed that both the 50 mg and 100 mg groups had a higher risk of AEs, with no significant difference in effect size between the two doses. Heterogeneity remained low in both subgroups.

Serious Adverse Events (SAEs)

**Figure 4:**
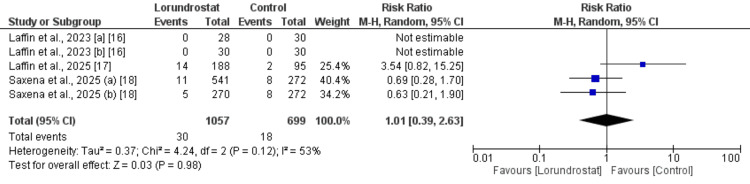
Comparison of serious adverse events between two groups [a] 50 mg dose; [b] 100 mg dose; blue square indicates risk ratio reported by individual study; black diamond indicates pooled estimate References [16-18]

Three studies were included to assess the risk of serious AEs, and the results of the pooled analysis are shown in Figure 4. The analysis showed no significant difference in the risk of serious AEs between the lorundrostat and placebo groups (RR: 1.01; 95% CI: 0.39 to 2.63). Moderate heterogeneity was observed across the studies (I^2^ = 53%).

Discussion

This systematic review and meta-analysis provide the first comprehensive evaluation of lorundrostat's efficacy and safety profile in patients with uncontrolled and TRH. Our findings demonstrate that lorundrostat therapy results in clinically meaningful reductions in SBP while being associated with an increased risk of AEs, though without a significant increase in SAEs compared to placebo.

The pooled analysis revealed that lorundrostat achieved a statistically significant reduction in SBP of 8.04 mmHg compared to placebo, with relatively low heterogeneity (I^2^ = 28%) among included studies. This magnitude of blood pressure reduction is clinically significant and aligns with established cardiovascular outcome benefits. According to landmark meta-analyses, every 5-mmHg reduction in SBP is associated with approximately a 10% reduction in major cardiovascular events [21,22]. The 8-mmHg reduction observed with lorundrostat therefore represents a potentially substantial cardiovascular protective effect, particularly relevant for the high-risk population of patients with TRH. The observed efficacy of lorundrostat reinforces the concept that aldosterone plays an important role in the pathogenesis of hypertension, extending beyond classical primary aldosteronism to encompass broader mechanisms of aldosterone-mediated blood pressure elevation [23,24].

The consistency of blood pressure-lowering effects across both 50 mg and 100 mg doses, as demonstrated in our subgroup analysis, suggests a relatively flat dose-response relationship within this dosing range. This finding has important clinical implications, as it indicates that the lower 50 mg dose may provide similar antihypertensive efficacy to the higher 100 mg dose, potentially optimizing the benefit-to-risk ratio. The absence of significant dose-dependent differences in efficacy may reflect the high selectivity and potency of lorundrostat for ASI, where maximal pharmacological effect is achieved at lower doses.

These results are particularly noteworthy when compared to traditional antihypertensive medications used as fourth-line therapy in TRH. The PATHWAY-2 trial, which established spironolactone as the preferred fourth-line agent, demonstrated a placebo-adjusted SBP reduction of 8.7 mmHg with low-dose spironolactone [25]. Our findings suggest that lorundrostat achieves comparable antihypertensive efficacy to spironolactone, representing a significant advancement given the novel mechanism of action and potential for improved tolerability profile.

Importantly, the absence of a significant increase in SAEs (RR: 1.01) provides reassurance regarding lorundrostat's overall safety profile. This finding is particularly relevant given historical concerns with ASIs, where earlier compounds demonstrated concerning effects on cortisol production and adrenal function. The lack of increased SAEs suggests that lorundrostat's high selectivity for CYP11B2 over CYP11B1 (selectivity ratio 374:1) successfully minimizes clinically significant cortisol suppression, addressing a key limitation that hindered the development of previous ASIs [8,17].

The pooled analysis revealed a significant increase in total AEs among participants receiving lorundrostat compared to placebo (RR: 1.47; 95% CI: 1.32 to 1.64), which requires careful interpretation in the context of the study populations and the nature of reported events. Across the included trials, the majority of AEs were classified as mild to moderate in severity. In the Target-HTN study, most AEs were deemed mild by investigators, with only 3-5% of participants in the lorundrostat groups experiencing severe AEs compared to 3% in the placebo group [16]. Similarly, in the Advance-HTN trial, severe AEs occurred in 5% and 11% of participants in the stable-dose and dose-adjustment groups, respectively, compared to 3% in the placebo group [17]. The Launch-HTN study demonstrated a comparable pattern, with severe AEs occurring in only 2% of participants across all lorundrostat groups versus 3% in the placebo group [18]. The observed increase in total AEs likely reflects the mechanism-based effects of aldosterone synthase inhibition, particularly predictable electrolyte disturbances such as hyperkalemia and modest effects on renal function, which are consistent with the pharmacological profile of blocking the renin-angiotensin-aldosterone system. Importantly, the predominance of mild-to-moderate events and the absence of unexpected safety signals suggest that the increased AE rate does not necessarily translate to clinically concerning safety issues, particularly when weighed against the substantial cardiovascular benefits associated with the observed blood pressure reductions.

Limitations and Future Directions

Several important limitations must be acknowledged. The analysis was constrained by the small number of included trials (n=3), reflecting lorundrostat's early clinical development stage. Follow-up durations were relatively short (4-12 weeks), limiting assessment of long-term efficacy, safety, and durability of blood pressure control. A critical limitation is the exclusive reliance on surrogate endpoints (SBP reduction) without cardiovascular outcome data. While the observed 7-9 mmHg reduction theoretically predicts cardiovascular benefits, this remains unproven for lorundrostat. Publication bias assessment was not feasible due to limited studies, representing a significant methodological concern given industry sponsorship of all trials. The moderate heterogeneity in the SAEs analysis suggests incomplete safety characterization. Additional limitations include the absence of head-to-head comparisons with established fourth-line therapies, particularly spironolactone, which would inform lorundrostat's positioning in resistant hypertension treatment algorithms.

## Conclusions

This meta-analysis provides promising early evidence that lorundrostat may represent a significant therapeutic advancement for patients with TRH. The demonstrated 8.04 mmHg reduction in SBP is clinically meaningful and comparable to established fourth-line therapies, with consistent efficacy across dosing regimens. While lorundrostat is associated with increased overall AEs, the predominance of mild-to-moderate events and absence of increased SAEs suggest a potentially acceptable safety profile. The drug's novel mechanism of aldosterone synthase inhibition addresses a key pathophysiological pathway in resistant hypertension while avoiding the compensatory aldosterone elevation seen with MRAs. However, given the limited number of studies and short follow-up durations, these findings require confirmation in larger, longer-term trials. Future research should prioritize long-term cardiovascular outcomes and direct comparative effectiveness studies to definitively establish lorundrostat's position in treatment algorithms for this challenging patient population.
